# eHealth literacy of migrant domestic workers in Hong Kong in the COVID-19 pandemic: A mixed methods study

**DOI:** 10.1371/journal.pone.0296893

**Published:** 2024-04-18

**Authors:** Ariesta Milanti, Dorothy Ngo Sheung Chan, Kai Chow Choi, Winnie Kwok Wei So

**Affiliations:** The Nethersole School of Nursing, Faculty of Medicine, The Chinese University of Hong Kong; National Taiwan University, TAIWAN

## Abstract

**Background:**

Health communication in the COVID-19 pandemic can be effectively implemented if all members of the populations, including marginalized population such as migrant domestic workers (MDWs), have good eHealth literacy. Lessons learned during this critical period may help improve planning and mitigation of the impacts of future health crises.

**Methods:**

This study aimed to examine and explore the eHealth literacy levels of the MDWs in Hong Kong during the COVID-19 pandemic by using a convergent mixed methods research design. A total of 1156 Hong Kong MDWs participated in a paper-based survey using a multistage cluster random sampling design for the quantitative component. eHealth literacy was measured using an eHealth literacy Scale (eHEALS). For the qualitative component, a purposive sampling of 19 MDWs participated in face-to-face, semi-structured, in-depth interviews. Descriptive statistics and multiple regression analyses were used to carry out the quantitative analysis, while thematic analysis was used for the qualitative analysis. Both quantitative and qualitative data were merged and integrated for mixed-methods analysis.

**Results:**

The meta-inferences of the quantitative and qualitative results mainly confirmed that MDWs in Hong Kong had good levels of eHealth literacy. The use of Instagram, YouTube and WhatsApp as the COVID-19 information sources, in addition to having an interest in the topic of the current spread of COVID-19 together with the Hong Kong government’s policies related to COVID-19, were found to be associated with eHealth literacy.

**Conclusions:**

The eHealth literacy level of MDWs in Hong Kong was shown to be good and it was influenced by the use of popular social media platforms including Instagram, YouTube, and WhatsApp. It is realistic to suggest that such platforms should be harnessed for health communication during the pandemic. Yet, regulations to combat false information on these media are also urgently needed.

## Introduction

The world has experienced a major global pandemic, namely the coronavirus disease 2019 (COVID-19) pandemic, which has resulted in nearly seven million deaths and over 700 million cumulative cases over a span of three years (2020–2023) [1]. While the pandemic has been officially declared over by the World Health Organization (WHO), it is important to reflect on the lessons learned during this critical period. The knowledge acquired can contribute to improve planning and mitigation of the impacts of future health crises, as well as safeguarding public health.

COVID-19 pandemic has underscored the importance of health communication for preventing infectious disease [2]. Health communication is integral to an effective public health response to an infectious disease outbreak [3]. Health communication encompasses the communication between health institutions, health professionals and the general public [4]. There are six main components that contribute to achieve effective health communication, namely: i) risk communication, ii) crisis communication, iii) outbreak communication, iv) health education, v) health advocacy, and vi) health literacy [5]. While the first five components focus on the communication efforts from the health authorities, institutions, or professionals, the last component, i.e. health literacy, is about the ability of individuals as members of the public.

Health literacy is “the extent to which individuals have the capacity to obtain, process, and understand basic health information and services needed to make appropriate health decisions” [6, 7]. In this COVID-19 pandemic eHealth literacy becomes highly relevant, since the internet has been the main platform for the dissemination and circulation of health information [8]. The common definition of eHealth literacy is basically similar to health literacy; however, the emphasis in the former is on the electronic sources of the health information [9]. People with good eHealth literacy are able to seek, find, understand, evaluate, and apply health information from the online sources [9]. These abilities are important means enabling an individual to understand and implement appropriate infectious disease preventive measures [10]. Previous studies found that people with higher eHealth literacy levels showed more adherence to COVID-19 preventive behaviors [8, 10, 11]. Without having the necessary eHealth literacy, when seeking and appraising information from the internet, people may fall into the trap of believing misinformation or disinformation which can impede their adherence to COVID-19 preventive measures or even lead to fatal consequences [2, 12, 13].

Health communication can be effectively implemented if all members of the populations are able to access, understand, and use the information being communicated [4]. It is important to note that in the COVID-19 pandemic, ‘the health of one is the health of all’ attitude should be applied [4]. Therefore, health communication efforts should address the needs of all populations, including marginalized elements such as migrant domestic workers (MDWs).

Hong Kong is a region in Asia which hosts a large number of MDWs [14]. At the end of 2021, there were a total of 339,451 MDWs in Hong Kong, of which 56.4% were from the Philippines, 41.2% were from Indonesia, and 2.4% were from other countries (including Sri Lanka, Thailand, and Nepal) [15]. MDWs work for around 337,700 households: 13.4% of all the households in Hong Kong [16]. MDWs in Hong Kong live with the employer families and typically perform caregiving, cleaning, and cooking tasks for that family [17].

MDW is one of the vulnerable, yet neglected, populations in the COVID-19 pandemic response [18]. Moreover, even in the non-pandemic situation, MDWs already encounter barriers in accessing health information and services, mainly due to language and cultural differences and their socioeconomic position [19]. Lower socioeconomic status, in particular, has been found to be associated with lower eHealth literacy [8, 20].

Previous studies conducted during the COVID-19 pandemic have examined eHealth literacy of health care workers [10], college / university students [21–25], and lay people [8, 26–28]. The majority of these studies found positive associations between eHealth literacy and COVID-19 preventive behaviors [8, 10, 25, 27, 29–32]. However, eHealth literacy of the more vulnerable and marginalized populations, such as MDWs, has not been studied. By focusing on the eHealth literacy of MDWs in Hong Kong during the COVID-19 pandemic, this study could address a significant gap in the literature and provide insights into their ability and specific challenges to access and utilize online health information effectively. Moreover, this study could also provide a unique perspective on a marginalized population, contributing to the broader understanding of eHealth literacy and its nuances. These insights could inform future research and policy development to ensure the effective delivery of health information for MDWs.

Therefore, the aims of this study were to examine and explore the eHealth literacy of the MDWs in Hong Kong. The following quantitative, qualitative, and mixed methods research questions (RQs) were used to achieve the study aims:

*Quantitative RQ 1*: What are the levels of eHealth literacy of the MDWs in Hong Kong?

*Quantitative RQ 2*: What are the factors associated with the Hong Kong MDWs’ levels of eHealth literacy?

*Qualitative RQ*: What are the eHealth literacy experiences of the MDWs in Hong Kong?

*Mixed methods RQ*: What do the quantitative and qualitative data reveal about the eHealth literacy of the MDWs in Hong Kong?

## Literature review

A highly dynamic situation such as COVID-19 pandemic usually comes with an infodemic, that is, is ‘the overabundance of information—some accurate and some not—that makes it harder for people to find trustworthy sources and reliable guidance when needed’[33]. This is one the main reasons why eHealth literacy is critically needed during the pandemic [33]. According to the WHO, eHealth literacy is the key to tackle the infodemic [33]. Several authors also argued that equipping people with the ability to obtain the correct and timely information, which is what eHealth literacy essentially means, was also one of the pillars in controlling the pandemic [34–36].

In a landmark paper on eHealth literacy, Norman and Skinner [9] proposed a model using the metaphor of a lily. eHealth literacy is depicted as a pistil of the flower, fed by six overlapping petals representing different literacies. The six literacies are categorized into two: analytic type (traditional, media, and information literacies) and context-specific type (computer, scientific, and health literacies) [9]. The analytic type of literacies encompasses basic skills needed for processing any kind of information sources in any context. Whereas, the context-specific literacies depend on a particular issue or setting.

The Lily model is widely accepted not only because it is a pioneering model in eHealth literacy area but also because it is simple yet encompasses the most fundamental elements of eHealth literacy [37]. However, this model has received several criticisms. Some authors, including Norman − who proposed the original Lily model, argued that this model is outdated, lack of contextualization and lack of communication/transactional nature of eHealth which is the prominent feature in the current ICT [37–41]. Indeed, the Lily model was developed in 2006 where the internet was dominated by texts created by web developers, before the rise of user- and artificial-intelligent (AI)-generated content as it is nowadays and in the near future.

Previous studies have demonstrated positive associations between some, but not all six, literacies in the Lily model and eHealth literacy itself. A recent systematic review supports the evidence showing the links of health literacy, computer literacy, and information literacy with the individuals’ eHealth literacy level [42]. Furthermore, the review [42] concluded that eHealth literacy is shaped by multilayered determinants, starting from the non-modifiable determinants (including age and sex). Studies have demonstrated conflicting findings regarding how age and sex influenced eHealth literacy [8, 26, 43–45]. However, more studies indicated that younger age and male sex are associated with higher eHealth literacy level [8, 26, 43]. Another individual layer affecting eHealth literacy is modifiable, including the literacy factors and online health information-seeking behavior factors [42]. In addition, the willingness to search health information and the intensity of internet use in general and Instagram use in particular are also associated with eHealth literacy [42]. Outside the individual layers, the environmental factors, i.e. the living and working conditions, can also determine eHealth literacy. Many studies consistently showed that higher education and income were associated with higher eHealth literacy (e.g. [8, 11, 43, 44]). Finally, language, which is situated in the outermost layer of eHealth literacy determinant, can also determine an individual’s eHealth literacy level [45].

**Fig 1:**
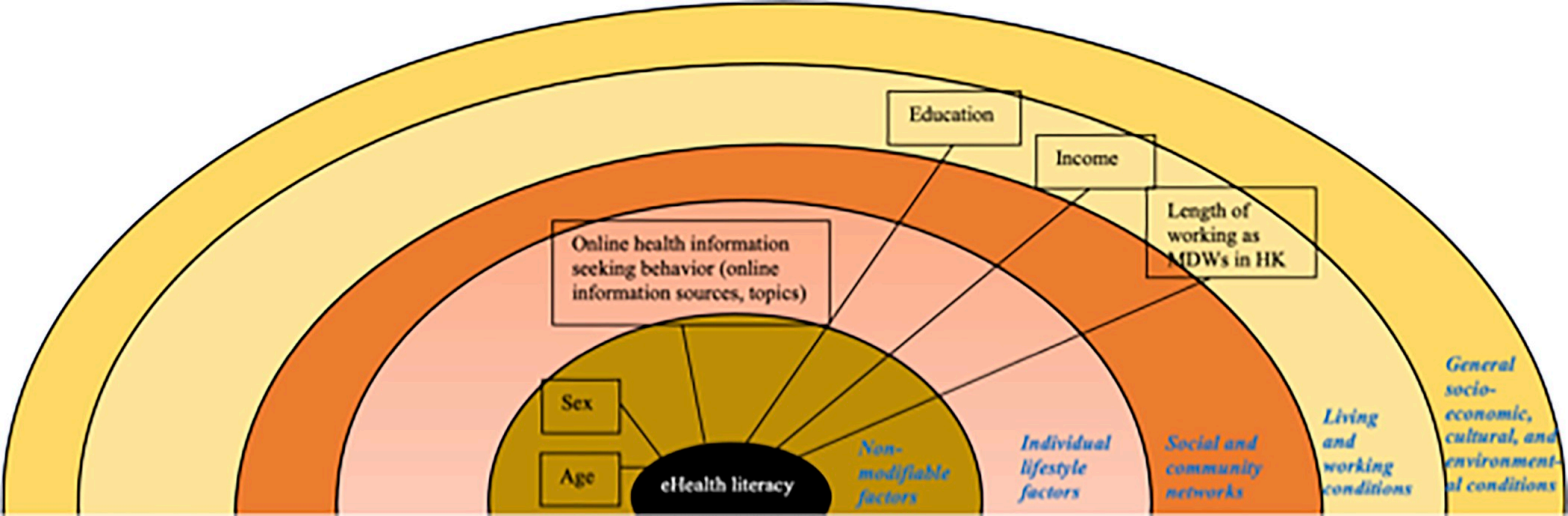
Conceptual framework of the study (modified from the eHealth literacy determinants model [42]).

This study adopted the conceptual model of eHealth literacy determinants outlined by Milanti et al. [42] and modified it to meet the study objectives. The conceptual framework is illustrated in Fig 1. Conceptual framework of the study (modified from the eHealth literacy determinants model [42]).

## Materials and methods

### Study design

This study used a convergent mixed methods design. Quantitative and qualitative data were collected and analyzed separately in a similar timeframe and then were merged for combination, comparison, or validation [46]. In the mixed methods design, the quantitative and qualitative methods could complement each other to obtain a thorough understanding of the topic. Using mixed methods design, robust evidence with rich insights could be obtained. Moreover, convergence and corroboration of the quantitative and qualitative results could be sought to enhance the validity of the study. In mixed methods research, numbers can be used to add precision to words, while words can be used to add meaning to numbers [47].

### Participants

As already stated above the population of this study is MDWs in Hong Kong. Inclusion criteria were as follows: (1) MDWs working in Hong Kong; (2) able to read and write in English or Bahasa Indonesia; (3) have a mobile phone and access to the internet; and (4) give the consent to participate in the study. Individuals who had cognitive impairment were excluded.

### The quantitative component of the study

The quantitative component of this study was a cross-sectional survey using a paper-based questionnaire. The minimum sample size was 1065 participants, calculated based on the estimated population size of the MDWs in Hong Kong which was 399,320 according to the HKSAR Immigration Department (2021) [48, 49]. A sample size of 1065 participants gives the study 90% power, at 5% level of significance, to detect a correlation as small as r = 0.1 in association analysis. The sample size estimation and power analysis were conducted using PASS 16.0 (NCSS, LLC. Kaysville, Utah, USA).

Data were collected by the researcher’s team, in person, by using a modified leisure place-based stratified probability sampling design, which was originally developed by Chung and colleagues [50]. During their rest days (typically Sundays), most MDWs in Hong Kong gather in the open public spaces, such as parks and footbridges, which are their leisure/catching-up meeting places. Chung, Liao, and Fong [50] have physically located and identified 125 ‘social hubs’ of the MDWs in 10 out of 18 districts in Hong Kong. The social hubs were categorized and color-coded as green (up to 10 MDWs), yellow (11–29 MDWs) and red (≥ 30 MDWs). The target number of participants was determined by the hub size (the color-coded hub), i.e. the green hub had 3 participants, the yellow hub had 6 participants and the red hub had 24 participants. A total of 8 green, 16 yellow and 40 red hubs were randomly selected within the sampling frame to achieve the target sample size of 1065 participants. The list of the social hubs was randomized using an online randomizer. Then, the participants were randomly selected in the selected social hubs, starting from the northernmost person to the data collectors followed by the alternate person in the clockwise direction until the target number of participants was met. The participants completed the questionnaires, which took them around 2–3 minutes, after signing the written informed consent.

The survey was divided into (a) demographic data, including the sources of online information and COVID-19-related topics of interest and (b) eHealth literacy. The demographic data included age, country of origin, marital status, education, income, and length of working in Hong Kong. Sources of online information and COVID-19-related topics of interest were assessed using a list adopted from a study by Dadaczynski et al. (2021).

eHealth literacy was assessed using an eHealth literacy scale/eHEALS [51]. eHEALS comprises eight items which examine the combined knowledge and perceived skills at finding, evaluating, and applying online health information to address health problems [51]. The eHEALS items were slightly modified to focus on COVID-19 online information, instead of general health information. eHEALS has a 5-point Likert scale so that the total score ranges from 8–40; the higher the score the higher is the respondent’s eHealth literacy level. eHEALS was selected because it is a valid and reliable instrument, as supported in the literature, and is also the most widely used eHealth literacy measuring instrument [52–59]. eHEALS has been translated for use with, and validated by, many populations including Indonesian [59]. In addition, eHEALS is short, simple, and easy to administer, hence it is suitable to target MDWs recruited in their leisure time and meeting places. eHEALS’s Cronbach’s alpha for this study was 0.949 (English) and 0.933 (Bahasa Indonesia).

Descriptive statistics and multiple regression analysis were performed in SPSS 25.0 (IBM Corp., Armonk, NY, USA). Independent variables including sociodemographic characteristics, sources of online information and topics searched were analysed using independent t-test and one-way ANOVA. Only independent variables with p < 0.25 in bivariate analyses [60] were selected as candidate independent variables for a backward multiple regression analysis. This process was adopted in order to identify the factors that are significantly and independently associated with eHealth Literacy score.

### The qualitative component of the study

The qualitative component of this study involved a descriptive approach. Participants were recruited using a purposeful sampling method. The inclusion criteria were similar to the criteria of the quantitative part, with an additional criterion of giving consent by any participant who took part in an audio-taped interview. The interview guide was developed based on the eHealth literacy definition which encompasses finding, evaluating, and using information from the internet [9]. The interview guide is presented in S1 File. Semi-structured, face-to-face, in-depth interviews were conducted by the first author until data saturation was reached. The Filipino participants were interviewed in English because they have good English proficiency and speak English daily at work. On the other hand, the Indonesian participants were interviewed in their native language (Bahasa Indonesia). The mean length of the interviews was 37 minutes 30 seconds.

Data were analyzed using a thematic analysis approach [61, 62]. The thematic analysis was done in six phases: (i) familiarization with the data, (ii) codes generation, (iii) themes construction, (iv) themes review, (v) themes revision, and (vi) producing a report [61]. To achieve rigor, prolonged engagement and triangulation, peer debriefing, negative case analysis, and member checking were all conducted; in addition, a reflexive journal was also kept.

After the separate analysis of the quantitative and the qualitative datasets, the data were merged to identify the converging and diverging findings. A joint display table was developed for presenting the side-by-side comparison of the quantitative and qualitative findings. The joint display provides a visual and transparent presentation of the data integration and interpretation in mixed methods studies [63]. The joint display table presented the key patterns and significant quantitative results alongside corresponding qualitative excerpts to illustrate the connections between the findings. Then, the merged findings were synthesized and interpreted to draw the meta-inference, i.e. the conclusions from the integration of the quantitative and qualitative evidence. The meta-inference process involved examining the extent to which the findings aligned or diverged and identifying overarching patterns that emerged from the integrated analysis.

Ethical approval for this study was attained from the Survey and Behavioral Research Ethics Committee of the Chinese University of Hong Kong (Reference No. SBRE-20-876). Participant recruitment, data collection, and data management were designed and executed in full recognition of the protection of the rights, dignity, and safety of those participants.

## Results

### Quantitative component

The data collection process took place from August until December 2021. A total of 1,461 MDWs were approached in the selected social hubs all over Hong Kong but 204 of them declined to participate in the study. A total of 1,257 MDWs filled in the questionnaires. Incomplete questionnaires (n = 101) were excluded, so finally 1,156 questionnaires were included for the data analysis.

The characteristics of the participants are summarized in Table 1. An overwhelming majority of the participants were female (99.2). The mean age of the participants was 36.60 years (SD = 7.86), with the majority (42.4%) being in the 31–40 years age group. Most participants were Indonesian (61.7%), were married (60.1%), went to senior high school (39.5%), had a monthly income of equal to or less than the minimum allowable wage (HK$ 4,630) (71.4%), and had been working in Hong Kong for > 5–10 years (32.4%).

**Table 1:** Characteristics of the participants (n = 1,156).

Characteristics	n (%)
**Gender**	
Female	1,147 (99.2)
Male	9 (0.8)
**Age group (years)**	
≤30	314 (27.2)
31–40	490 (42.4)
41–50	302 (26.1)
>50	50 (4.3)
**Country**	
Indonesia	713 (61.7)
Philippines	443 (38.3)
**Marital status**	
Married	695 (60.1)
Single/Not married/Divorce	461 (39.9)
**Latest education**	
Elementary school	85 (7.4)
Junior high school	359 (31.1)
Senior high school	457 (39.5)
College/University	255 (22.1)
**Monthly income (HK$)**	
≤4,630	825 (71.4)
>4,630	331 (28.6)
**Length of working in Hong Kong**	
≤3 years	307 (26.6)
>3–5 years	260 (22.5)
>5–10 years	375 (32.4)
≥10 years	214 (18.5)

Nearly all participants (97.1%) sought and obtained COVID-19 information from online sources (Table 2). The most frequently used information source was Facebook (93.8%) and the least frequently used online source was Twitter (15.9%).

**Table 2:** Sources of online information and topics of interest (n = 1,156).

Variable	n (%)
**Online information-seeking behaviour**	
Getting information from online sources	1,123 (97.1)
**Sources of information** ^**a**^	
Facebook	1,084 (93.8)
Instagram	442 (38.2)
WhatsApp	740 (64.0)
YouTube	792 (68.5)
TikTok	330 (28.5)
Twitter	184 (15.9)
Website	430 (37.2)
**Topics of interest**	
Signs and symptoms of COVID-19	1,087 (94.0)
Government policies related to COVID-19	1,080 (93.4)
COVID-19 preventive measures	1,079 (93.3)
Current spread of COVID-19	1,070 (92.6)
Other (Vaccination)	28 (2.4)

^a^ Participants could choose more than one source of information.

The mean value of eHEALS score was 32.28 (SD = 4.14). With regards to the factors associated with eHealth literacy, using Instagram (B = 0.69, 95% CI 0.19–1.20, *p* value = 0.007), WhatsApp (B = 0.67, 95% CI 0.16–1.18, *p* value = 0.010), and YouTube (B = 0.60, 95% CI 0.07–1.12, *p* value = 0.026) as the sources of information were associated with higher eHealth literacy (Table 3). Furthermore, having an interest in the topics relating to COVID-19’s current spread (B = 1.82, 95% CI 0.86–2.78, *p* value < 0.001) and related government policies (B = 1.05, 95% CI 0.03–2.07, *p* value = 0.043) were also identified as positive factors enhancing eHealth literacy.

**Table 3:** Factors associated with eHealth Literacy (n = 1,156).

Variables	Bivariate analysis		Multivariate analysis	
	Mean (SD)	p value	B (95% CI)	p value
**Demographic characteristics**				
Age (years)				
≤30	32.70 (3.83)	0.027	NS	
31–40	32.35 (3.96)			
41–50	31.93 (4.42)			
>50	31.16 (5.55)			
Gender				
Female (ref)	32.37 (4.14)	0.212	NS	
Male	34.00 (4.27)			
Country of origin				
Indonesia (ref)	32.35 (3.67)	0.527	NE	
Philippines	32.18 (4.80)			
Marital status				
Married (ref)	32.23 (3.69)	0.602	NE	
Single/Not married/Divorced	32.37 (4.74)			
Education attainment				
Elementary school (ref)	31.22 (4.47)	0.041	NS	
Junior high school	32.22 (3.79)			
Senior high school	32.59 (3.98)			
College/University	32.19 (4.69)			
Income per month (HK$)				
≤4,630 (ref)	32.21 (3.83)	0.355	NS	
>4,630	32.48 (4.18)			
Years of working in Hong Kong				
≤3 (ref)	32.55 (4.03)	0.290	NE	
>3–5	32.49 (3.69)			
>5–10	32.02 (4.12)			
>10	32.10 (4.69)			
**Online information-seeking behaviours**				
*Sources of COVID-19 information*				
Facebook				
No (ref)	30.47 (4.97)	0.002	NS	
Yes	32.40 (4.05)			
Instagram				
No (ref)	31.84 (3.93)	<0.001		
Yes	33.01 (4.36)		0.69 (0.19–1.20)	0.007
WhatsApp				
No (ref)	31.55 (4.86)	<0.001		
Yes	32.70 (3.61)		0.67 (0.16–1.18)	0.010
YouTube				
No (ref)	31.48 (4.54)	<0.001		
Yes	32.65 (3.89)		0.60 (0.07–1.12)	0.026
TikTok				
No (ref)	31.99 (4.18)	<0.001	NS	
Yes	33.03 (3.95)			
Twitter				
No (ref)	32.16 (4.20)	0.017	NS	
Yes	32.95 (3.73)			
Website				
No (ref)	32.16 (4.17)	0.198	NS	
Yes	32.49 (4.07)			
*Topics of COVID-19 information*				
Current spread of COVID-19				
No (ref)	29.81 (4.38)	<0.001		
Yes	32.48 (4.05)		1.82 (0.86–2.78)	<0.001
Signs and symptoms of COVID-19				
No (ref)	30.00 (4.49)	<0.001	NS	
Yes	32.43 (4.07)			
Preventive measures				
No (ref)	30.38 (5.003)	0.001	NS	
Yes	32.42 (4.09)			
Government policies related to COVID-19				
No (ref)	30.17 (4.53)	<0.001	1.05 (0.03–2.07)	0.043
Yes	32.43 (4.07)			

B: regression coefficient; CI: confidence interval; ref: reference category of categorical variable.

NE: not entered into the backward multivariable regression analysis; NS: not significant.

### Qualitative findings

Nineteen MDWs participated in the individual semi-structured interviews. The summary of the participants characteristics is presented in Table 4. The mean age of the participants was 36.42 years (SD = 7.48). The majority of the participants were female, married, originated from Indonesia and had completed senior high school. The mean eHealth literacy score of the participants was 34 (SD = 4.59). [Table 4]

**Table 4:** Participants characteristics of the qualitative component.

Participant code	Gender	Age	Education	Length of working in Hong Kong	MaP7l status	Country	Monthly income (HK$)*	eHEALS score
P1	Female	25	Junior high school	1.91 year	Single	Indonesia	4,630	32
P2	Female	42	College/University	6 years	Married	Indonesia	5,000	38
P3	Female	37	Senior high school	8 years	Married	Indonesia	4,630	40
P4	Female	30	Senior high school	10 years	Single	Indonesia	4,630	32
P5	Female	52	Junior high school	12 years	Married	Indonesia	4,630	30
P6	Female	39	Senior high school	9 years	Single	Indonesia	4,630	40
P7	Female	40	College/University	8 years	Married	Indonesia	4,630	32
P8	Female	34	Senior high school	7 years	Single	Indonesia	4,700	30
P9	Male	40	College/University	5 years	Married	Indonesia	15,000	40
P10	Female	31	Junior high school	5.75 years	Single	Indonesia	4,630	12
P11	Female	34	Senior high school	5.50 years	Married	Indonesia	4,630	32
P12	Female	23	Senior high school	2 years	Single	Indonesia	5,000	40
P13	Female	50	College/University	20.33 years	Married	Philippines	4,630	40
P14	Female	38	College/University	9.58 years	Single	Philippines	4,630	32
P15	Female	37	College/University	10.91 years	Married	Philippines	6,000	38
P16	Female	36	Junior high school	3.75 years	Single	Philippines	4,630	32
P17	Female	27	Senior high school	3.33 years	Single	Indonesia	4,630	32
P18	Female	35	Senior high school	9.83 years	Married	Philippines	4,630	32
P19	Female	42	College/University	9.91 years	Married	Philippines	5,000	32

*1 HK$ = 7.8 USD

Six themes were identified in this study. The themes were: 1) process of getting COVID-19 information, 2) evaluating COVID-19 information, 3) barriers to eHealth literacy, 4) facilitators of eHealth literacy, 5) loopholes in eHealth literacy, and 6) outcomes of eHealth literacy.

#### Process of getting COVID-19 information

The participants described the process of obtaining COVID-19 information, starting from hearing about the information from their employer, family, or friends. Then they would ‘*just Google it*,’ choose the sources among many sources available on Google, and choose what to read. Afterwards, they would continue reading and monitoring the information from the sources that they trusted to meet their information needs about COVID-19. For example, one participant narrated how she went through the process of COVID-19 information seeking, while acknowledging the existence of false information. She therefore emphasized the importance of choosing the information worth-reading.

‘Yes, go to Google, and then choose, what you like to see. But in Google sometimes, the top searches, if it is about the question that you would like to Google, the top searches you have to choose there. But there are so many conspiracy things. But you have to choose, which one you like to read and which one you like to understand’. (P13)

#### Evaluating COVID-19 information

The MDW participants shared their experiences in evaluating COVID-19 information obtained from electronic sources, an essential element of eHealth literacy. The majority of participants mentioned that the most important and at the same time the entry point of the evaluation is by choosing the credible source. The participants said that they filter the information by the credibility of the source, as illustrated in the following quote.

‘For me, my first filter is to get the information from reliable sources’. (P6)

Some participants also compared the information from various sources to check its credibility levels. In addition, they used logic and critical thinking, and also sought details about the information sources and the authors. These were ways to evaluate the accessed information, as described in the following quotes.

‘So, if we, for example, are looking for information in other sites, we have to choose, we have to filter, don’t take everything into our mind, don’t just take the information, we have to think which one is logical and which one is not.’ (P17)

‘Maybe other people believe easily. But for me, I will search as much detail as I can.’ (P12)

#### Barriers to eHealth literacy

Seven Indonesian participants mentioned that language is a barrier that can hinder the search for, or understanding of, online information about COVID-19. Many Indonesian MDWs in Hong Kong have low levels of English proficiency. Therefore, for accessing English COVID-19 information, they need to use the translation service provided by the internet platform. However, if the translation platform does not help, they need to rely on the information sources presented in Bahasa Indonesia. For example:

‘In Google, sometimes I get the information in English or Mandarin, which will make me blank, I have no idea. I try to put it on Google Translate, but sometimes it is still vague. So I will look for information in Bahasa Indonesia’. (P9)

It should be noted that the vast majority of Filipino MDWs understand English and have no problem in seeking and obtaining COVID-19 information presented in the medium of English.

According to some participants, another barrier to eHealth literacy is the MDW’s heavy workload, which greatly limits the time they have to explore the news on the internet. In addition, two different situations on information availability need to be taken into account: i) there was too little information about COVID-19 at the beginning of the pandemic and ii) currently there is far too much information. Both these factors were perceived by participants barriers to eHealth literacy.

#### Facilitators of eHealth literacy

Despite the barriers to eHealth literacy, there were two strong facilitators of eHealth literacy of the MDWs in Hong Kong: 1) easy internet access in Hong Kong and 2) everyone owns a smartphone. In Hong Kong, an internet data plan, which is the major source of internet access for the MDWs, is affordable and provides fast internet access.

‘I think it is quite flexible. It is quite easy. First, maybe because the internet access here is cheap. We can use it to search for anything available online that we need and it is accurate’. (P4)

Furthermore, the ownership of a smartphone is also a facilitator of eHealth literacy. Nowadays, everyone, including the MDWs, not only has a mobile phone but a smartphone to easily access internet and mobile applications.

#### Loopholes in eHealth literacy

Even though most participants demonstrated good eHealth literacy levels, loopholes existed in the eHealth knowledge of a few participants. It was also suggested by some participants that their ability in seeking and evaluating COVID-19 from electronic sources (as shown in the previous themes) caused them to have some difficulties in differentiating between accurate and inaccurate information.

‘Aargh, difficult! You will not know who says (it is) correct, who says this is wrong. Because if other people learn from this, they will follow also. They will say “oh this one saying (it) is correct, and this one is wrong.” So, we need to follow that. So, we don’t know who to follow…this one or that one or this one’. (P16)

One participant, the same participant who mentioned, ‘but I don’t follow all the news, only the important ones, because I have to work’, also mentioned that sometimes she did not really understand what she read. This scenario implies a loophole in eHealth literacy, in that she might only find and read the information, without really understanding and critically appraising it. Such a challenge can create a confusing and potentially dangerous situation:

‘I just saw. Just saw. Some news. I just read, but I don’t really understand’. (P5)

#### Outcomes of eHealth literacy

The participants described that as the results of having good eHealth literacy, they could make their own health decisions, especially regarding the issue of vaccination.

‘Yeah! (I) did my own research to decide for myself’. (P18)

Some of the participants also changed their health behavior as they learned from the online information sources.

‘…We did not wear masks at that time (the beginning of the pandemic). But then we learned from YouTube, Facebook, and browsed from other sources on the internet and also from friends. Then, we started wearing masks.’ (P7)

Furthermore, having appropriate levels of eHealth literacy was found to be beneficial not only for self-education, but also for educating others, such as the MDWs’ family member(s) back in the home country or her/his friends. In addition, MDWs also mentioned that having eHealth literacy has helped them to get practical information that they need; for example: how to get a vaccination in the community vaccination centers.

### Mixed methods findings

The quantitative and qualitative findings were compared and merged based on the eight domains of eHealth literacy Scale (eHEALS) and are presented in a joint display (Table 5). Meta-inferences were drawn from the comparison between quantitative and qualitative component results. In six domains of eHealth literacy, i.e.

1. *I know what COVID-19 resources are available on the internet*,
2. *I know where to find helpful COVID-19 resources on the internet*,
3. *I know how to find helpful COVID-19 resources on the internet*,
4. *I know how to use the internet to answer my questions about COVID-19*,
5. *I have the skills to evaluate the COVID-19 information found on the internet*,
6. *I can tell high-quality COVID-19 information from low-quality ones on the internet*,

the qualitative findings confirm the quantitative findings. Most of the participants in the quantitative and qualitative components reported that they had the above mentioned knowledge and skills. In two domains (*I know how to use COVID-19 information I find on the internet to help me* and *I feel confident in using COVID-19 information from the internet to make health decisions)*, the qualitative findings expand the results of the quantitative component. For example, the participants not only felt confident in using the online information to make health decisions for themselves, but also felt able to educate other people about COVID-19. Nevertheless, in domain number 7, regarding evaluating high-quality COVID-19 information, there is also a discordant meta-inference which corresponds to the theme ‘loopholes in eHealth literacy’. Even though the majority of the participants reported that they could evaluate COVID-19 information, a few participants in the qualitative component described how they still had difficulty in appraising the validity and merit of the COVID-19 information that they obtained from the internet. [Table 5]

**Table 5:** Joint display of eHealth literacy domains.

Domain	Quantitative (n = 1,156)	Qualitative theme and selected quotes	Meta-inference
*1. I know what COVID-19 resources are available on the internet*	Agree = 832 (72.0%) Strongly agree = 258 (22.3%) (total agree and strongly agree = 1090 (94.3%)) Undecided = 42 (3.6%) Disagree = 10 (0.9%) Strongly disagree = 14 (1.2%)	***Process of getting COVID-19 information*** ‘Yeah. YouTube is the largest platform. When you watch there, you can find some blogs from the doctors that introduce you about the vaccine, introduce you about the virus. So you can study also, you can learn from them what is right and what is wrong. How can you survive from this pandemic. Actually YouTube is nice’. (P16)	***Confirmation*** Knowing what COVID-19 resources available on the internet is a part of the process undertaken by MDWs to get COVID-19 information. The MDWs mentioned general platform, and some of them mentioned specific resources such as from the government or the news outlet.
*2. I know where to find helpful COVID-19 resources on the internet*	Agree = 874 (75.6%) Strongly agree = 196 (17.0%) (Total agree and strongly agree = 1070 (92.6%)) Undecided = 64 (5.5%) Disagree = 12 (1.0%) Strongly disagree = 10 (0.9%)	***Process of getting COVID-19 information*** ‘I go to government websites, such as SCMP and RTHK. They don’t have all the news, but I’m sure their news is accurate’. (P4)	***Confirmation*** The MDWs stated that they knew where to find COVID-19 resources on the internet. They would usually have their list of resources that they trust and prefer. Most of the MDWs mentioned government sources (especially Facebook page and website) as helpful COVID-19 resources. Other sources were local or international news outlets.
*3. I know how to find helpful COVID-19 resources on the internet*	Agree = 853 (73.9%) Strongly agree = 211 (18.3%) (Total agree and strongly agree = 1064 (92.2%)) Undecided = 70 (6.1) Disagree = 15 (1.3) Strongly disagree = 7 (0.6)	***Process of getting COVID-19 information*** ‘Yes, go to Google, and then choose what you like to see. But in Google sometimes, the top searches, if it is about the question that you would like to google, the top searches you have to choose there. But there are so many conspiracy things. But you have to choose, which one you like to read and which one you like to understand’. (P13)	***Confirmation*** The MDWs showed their capability in finding helpful COVID-19 resources on the internet, while distinguishing for the unhelpful information. They knew how to get into reliable COVID-10 resources by searching on Google search engine.
*4. I know how to use the internet to answer my questions about COVID-19*	Agree = 837 (72.4%) Strongly agree = 204 (17.6%) (Total agree and strongly agree = 1041 (90%)) Undecided = 97 (8.4%) Disagree = 11 (1.0%) Strongly disagree = 7 (0.6%)	***Process of getting COVID-19 information*** ‘…Facebook, internet, just *google* it right?’ (P19) ‘The first thing I do when I dig up information, whatever information, I will look it up on Google, on the internet. It will answer anything that I would like to dig or I’m curious about’. (P11) ‘The key words are: update + covid + Hong Kong’. (P4)	***Confirmation*** The MDWs showed their familiarity with using internet to search for information. They used social media platforms on a daily basis, and they mostly used Google search engine to answer their questions related to COVID-19 or any other issues. They could delineate important key words to address their information need.
*5. I know how to use COVID-19 information I find on the internet to help me*	Agree = 843 (72.9%) Strongly agree = 196 (17.0%) (Total agree and strongly agree = 1039 (89.9%)) Undecided = 100 (8.7%) Disagree = 8 (0.7%) Strongly disagree = 9 (0.8%)	***Outcomes of eHealth literacy*** ‘Now I remember. There is a government site about COVID-19, where you can get the vaccine, the community…which community you can get the vaccine, the scheduling, the booking, and everything about COVID-19 information’. (P14) ‘I’m on Facebook just to give information and education for my fellow migrant domestic workers’. (P17)	***Expansion*** The MDWs stated that they knew how to utilise the information they found online to help them with practical issues. As an expansion, they also acknowledged other benefits of obtaining COVID-19 online information for educating themselves and other MDWs.
*6. I have the skills to evaluate the COVID-19 information found on the internet*	Agree = 780 (67.5%) Strongly agree = 164 (14.2%) (Total agree and strongly agree = 944 (81.7%)) Undecided = 191 (16.5%) Disagree = 16 (1.4%) Strongly disagree = 5 (0.4%)	***Evaluating COVID-19 online information*** ‘I usually evaluate the accuracy of an information by seeing the source, the credibility of the source’. (P8) ‘Because I will collect the information not only from 1 to 2 sources, but from 3 to 4 sources. I will collect and compare. That’s how I observe the information’. (P12)	***Confirmation*** The MDWs narrated their experiences in using several ways to evaluate the COVID-19 online information, ranging from using the critical judgement to utilising their social network. Obtaining information from reliable sources is the first filter that they used for evaluating online information. Comparing references, using logic, researching the details, and having follow-up discussion were other ways for examining the accuracy of COVID-19 information found on the internet.
*7. I can tell high-quality COVID-19 information from low-quality ones on the internet*	Agree = 764 (66.1%) Strongly agree = 185 (16.0%) (Total agree and strongly agree = 949 (82.1%)) Undecided = 187 (16.2%) Disagree = 15 (1.3%) Strongly disagree = 5 (0.4%)	***Theme 1*: *Evaluating COVID-19 online information*** ‘I said earlier that I look for the information from the credible sources. So if I have taken the information from the government website, automatically the information has gone through the procedures to reach the public. Government would not tell fake news, right? That’s the way I evaluate the information. But it does not mean that everything that the government says is true’. (P3) ***Theme 2*: *Loopholes in eHealth literacy*** ‘For example, there are some information which makes me think: “really?”, it seems untrue, but it is difficult to get the information which are really accurate. That’s the difficulty. Even I could say that sometimes, I would just become sceptical to all information available. I still need to think, is it true or not? To choose the information is also difficult, to see which one is current and which one is not. Because hoax and truth are difficult to differentiate’. (P1)	***Confirmation*** The MDWs mentioned that they know how to differentiate the high-quality information from the low-quality ones. They used the same ‘first-line filter’ for evaluating information, that is, choosing only the reliable sources, such as government websites. Other ways to evaluate COVID-19 online information were also aimed at obtaining high-quality information. ***Discordance*** Although the majority of the MDWs in the quantitative and qualitative components showed that they could tell high-quality COVID-19 online information from the low-quality ones, a few anomaly cases were revealed in the qualitative part. A few participants expressed their difficulties in differentiating between fake and real news.
*8. I feel confident in using COVID-19 information from the internet to make health decisions*	Agree = 820 (70.9%) Strongly agree = 198 (17.1%) (Total agree and strongly agree = 1018 (88%)) Undecided = 101 (8.7%) Disagree = 25 (2.2%) Strongly disagree = 12 (1.0%)	***Outcomes of eHealth literacy*** ‘Yeah, did my own research to decide for myself’. (P18)	***Confirmation*** The MDWs stated that they were confident in using COVID-19 online information to make health decisions related to COVID-19, especially vaccination which is done on a voluntary basis. The information that they found on internet made them feel empowered as they could make their own decisions and not depend on their employers. ***Expansion*** In addition, changing health behaviours is also the outcome of eHealth literacy which shows the MDWs’ confidence in using the COVID-19 online information.

## Discussion

This mixed methods study offers a comprehensive understanding of the eHealth literacy of the MDWs in Hong Kong. This study found converging evidence, in which quantitative findings were confirmed by the qualitative findings: *MDWs in Hong Kong possessed a good level of eHealth literacy*.

The mean score of eHealth literacy of the MDWs in this study is 32.28 out of 40 (SD = 4.14). This score is higher than the mean eHEALS scores in most of eHealth literacy studies. For example, the mean eHEALS score of MDWs in this study is higher than those of American internet users (mean = 29.0) [27], Japanese internet users (mean = 23.4) [64], nurses in South Korea (mean = 28.21) [65], and lay people in Hong Kong (mean = 26.10) [8]. Good eHealth literacy was also identified in the experiences of MDWs in dealing with COVID-19 information from the online sources. MDWs described that they were capable of seeking, understanding, and evaluating COVID-19 information from electronic sources. Moreover, they could also apply the useful online information they found on the internet to make health decisions and change health-focused behaviors.

That MDWs had a high level of eHealth literacy is an interesting finding since it disagrees with the previous study findings that people with lower socioeconomic status were more likely to have a lower level of eHealth literacy [8, 21]. This finding also answers the skepticism about the MDWs’ eHealth literacy; a point which was raised by a previous study [66].

A plausible explanation for this phenomenon can be taken from the qualitative finding of the ‘facilitators of eHealth literacy.’ According to the MDWs themselves, their ownership of a smartphone, and having easy internet access, greatly facilitated their eHealth literacy. MDWs in Hong Kong can enjoy the well-developed information and communication technology infrastructure and mobile data services which are among the world’s fastest [67]. In addition, for most MDWs in Hong Kong, they can access their mobile phone in between their work, or after work, without unduly strict limitations from their employers [68]. By contrast, many live-in MDWs in Japan, Malaysia, and Singapore are forbidden by their employers from using their mobile phones during work time [69]. It has been suggested that individuals who frequently engage in the search and interpretation of online information are more likely to have more confidence to do so than less frequent users [70]. Having good internet access and familiarity with mobile devices are two factors that are also positively associated with eHealth literacy [71].

Another possible explanation of the high eHealth literacy level of the MDWs is because this study was carried out in the context of the COVID-19 pandemic. The eHealth literacy questions in the survey and interview focused on COVID-19 information. The data for this study was collected in late 2021, approximately 1.5 years after the beginning of the pandemic, so the MDWs had 18 months to have accumulated the information regarding COVID-19 from the internet.

However, the mixed methods finding of this study showed that there was a discordance between the eHealth literacy results of the quantitative and qualitative components of the research, as can be seen in the theme ‘loopholes in eHealth literacy.’ While the majority of the survey participants reported a good level of eHealth literacy, there were four participants in the qualitative component who struggled to differentiate between accurate and inaccurate information. This anomaly might indicate the intricacies and challenges of finding reliable information on the internet regarding and during this pandemic. Most participants of this study were social media users. Studies showed that trustworthy sources are significantly under-represented on the social media [72, 73]. A huge portion of information on the social media is user-generated and thus the contents are often mixed with subjective or inaccurate information [73]. Therefore, it takes high quality eHealth literacy of the individuals to assess the accuracy of the information. The discordant result of this present study suggested that there is a small population of MDWs, with lower levels of eHealth literacy, that needs more attention and supportive intervention for future studies.

In addition, there were ‘expansion’ results in the mixed methods findings, regarding using the online information ‘to help myself’ and ‘to make health decisions.’ Some MDWs in the qualitative component stated that they did not only use the information from the internet for themselves but also for other people, especially their family back home and other MDWs. This finding reveals the social dimension of eHealth literacy. Previous studies also found that eHealth literacy was positively associated with social support and interpersonal relationships [65, 74]. By having/acquiring eHealth literacy, people can provide or exchange health information which might benefit and strengthen their social network [75]. With regards to the health decision making (eHEALS domain number 8), the qualitative component showed that eHealth literacy’s outcome was more than decision making but also involved changing health behaviors. Indeed, accumulating evidence suggests there exists a link between eHealth literacy and health promoting behaviors in general, as well as COVID-19 preventive behaviors in particular [8, 10, 25, 27, 29–32].

Using Instagram, WhatsApp, and YouTube as the sources of online COVID-19 information was found to be positively associated with eHealth literacy. On the other hand, there was no statistically significant relationship between the use of Facebook, Twitter, TikTok, website, and eHealth literacy. Conversely, the results of previous studies done among Slovenian and Danish university students found that those with a good level of eHealth literacy were more likely to use websites as their information source, while students with a lower level of eHealth literacy tended to seek information from the social media [22, 76].

WhatsApp can be more susceptible to misinformation since it does not have the algorithm to counter such unhelpful material [77, 78]. Moreover, information shared on WhatsApp may be more convincing because it is shared by people with strong social networking ties and the content may touch on users’ affective concerns and personal experiences [79]. MDWs using WhatsApp as their COVID-19 online information source may need to apply more of their eHealth literacy skills, particularly on evaluating the accuracy of the information. Instagram and YouTube, on the other hand, have their own mechanisms to manage false information, yet misinformation still spreads widely on these platforms [80]. Therefore, MDWs also need to carefully evaluate the information obtained from these media sources. MDWs using these platforms may also use the internet more often, as they are immersed in the contents or feeds offered by Instagram and YouTube. A previous study indicated that greater internet use is positively associated with greater eHealth literacy levels [81].

Regarding the topics of interest related to COVID-19, this study found that having an interest in the topic of ‘the current spread of COVID-19’ and ‘government policies related to COVID-19’ were positively linked to higher levels of eHealth literacy. Meanwhile, having an interest in the topics of ‘signs and symptoms of COVID-19’ and ‘prevention measures of COVID-19’ were not statistically significantly associated with eHealth literacy. A study by Zakar [82] found that the current spread of COVID-19 and the symptoms of COVID-19 were the dominant topics sought by university students in Pakistan. However, the researchers did not assess their respondents’ associations with eHealth literacy. Hong Kong MDWs who had an interest in the topic of the current spread of COVID-19 and government policies related to COVID-19 might need more eHealth literacy skill to seek and update this information, which changed from time to time as it was updated. On the other hand, the topics ‘sign and symptoms of COVID-19’ and ‘prevention measures’, which tended to remain unchanged, might explain why they were not associated with enhanced eHealth literacy.

The main limitation of this mixed methods study is that the sampling method was not ‘nested’ sampling, which might have allowed more MDWs with low eHealth literacy from the quantitative component to be recruited for the qualitative component. Recruiting participants with low eHealth literacy was challenging. In the quantitative part, only 2% of the total sample (23 out of 1156 participants) had low eHealth literacy levels. Furthermore, in the qualitative part of this study, there was only one participant with a low eHealth literacy score. These numbers illustrate a consistently small proportion of the Hong Kong MDWs with a low eHealth literacy level. Nevertheless, in the qualitative data analysis, we put a high sensitivity to the cases of low eHealth literacy. Future studies can focus on MDWs with low eHealth literacy levels and identify strategies to improve their eHealth literacy.

This study reveals several practical implications. Health communication could be done by harnessing social media platforms including Instagram and YouTube and a social messaging platform such as WhatsApp. However, regulations to effectively govern false information on these platforms is urgently required.

In this study, we have listed the most popular platforms as sources of health information during the COVID-19 pandemic based on the literature. We also emphasized the significance of social media platforms due to their profound influence on public perceptions and behaviors. Conversely, the term ’website,’ which can encompass a vast array of websites, from government websites to individual-run blogs, was not specified in the quantitative part of the study due to its impracticality. Thus, we addressed this limitation through the interviews by exploring participants’ choices of websites during the COVID-19 pandemic. Future studies can examine the wide array of online resources to which eHealth literacy, as a set of perceived skills, should be adaptable.

Furthermore, one of the meta-inference ‘expansion’ results showed that MDWs with higher eHealth literacy could educate not only themselves but also other MDWs. Thus, future studies can develop peer interventions for improving the health behavior of the MDWs. Health communication can also be strengthened by involving influencers within the MDW communities. The influencers can help tailor the health information to be more linguistically and culturally appropriate for the MDW population.

Conducting research during the COVID-19 pandemic revealed valuable lessons, one of which is the difficulty of keeping pace with the rapidly changing situation of the pandemic. For example, one part of our study that was planned to assess the critical topics in early 2021 were already deemed less important by the time data collection took place in late 2021. This experience underscored the importance of adaptability and responsiveness in research. The dynamic nature of the pandemic required the agility of the researchers to timely capture the most salient findings of the phenomenon under study.

## Conclusions

This study provides evidence that during the COVID-19 pandemic MDWs in Hong Kong had good levels of eHealth literacy. Several factors linked to eHealth literacy were identified: the use of Instagram, YouTube and WhatsApp as the COVID-19 information sources, and having an interest in the topic of the current spread of COVID-19 and the Hong Kong’s government policies related to COVID-19. The researchers suggest that social media platforms could and should be harnessed for health communication during the pandemic. However, regulations are needed to combat the misinformation and disinformation that comes from these platforms.

## Supporting information

S1 File Interview guide. (DOCX)
