# Supplementary material for: eHealth literacy of migrant domestic workers in Hong Kong in the COVID-19 pandemic: A mixed methods study
Source: PLoS One. 2024 Apr 18;19(4):e0296893. doi: 10.1371/journal.pone.0296893 (PMC11025963; doi:10.1371/journal.pone.0296893)
Supplement: S1 File — (DOCX) [file pone.0296893.s001.docx]

# Supporting information

**S1. Interview guide**

| Question | Probe |
| --- | --- |
| 1. Please tell me about your experiences in finding helpful information about COVID-19 on the internet? | - Why do you seek information about COVID-19? - How did you obtain online information about COVID-19 in the early time of the pandemic? How about nowadays? - What media do you use most frequently (social media, e.g. Facebook, TikTok, Instagram, YouTube; mobile application, e.g. WhatsApp; website, etc)? Other media? - What topics of COVID-19 information do you seek or obtain? - How confident are you in finding useful and accurate COVID-19 information on the internet? |
| 1. How do you know if the information you get from the internet is accurate or not? | - How confident are you in checking the accuracy of the COVID-19 information found on the internet? - Have you ever come across hoaxes or wrong information on the internet? - What are the hoaxes that you have come across? - How can you tell if an information is a fact or a hoax? - Do you check the accuracy of the information before sharing it? - How do you check the accuracy? |
| 1. Could you please tell me about your experiences in using the information about COVID-19 prevention that you get from the internet? | - How confident are you in using COVID-19 information that you find on the internet? - How did you learn about the recommendation of wearing a mask? How do you adhere to it? - How did you learn about the recommendation of cleaning your hand? How often do you clean your hand? When will you wash your hand at home? Do you keep a hand sanitiser when you’re going out? - How did you learn about the recommendation of keeping rooms well ventilated? Do you apply it in your own room (if there is any)? How about in the house? - How did you learn about the recommendation of coughing into a bent elbow or tissue? How do you adhere to it? - How did you learn about the recommendation of physical distancing? How do you adhere to it? - How did you learn about the recommendation of avoiding crowds? How do you adhere to it? - How did you learn about the recommendation of vaccination? Have you been vaccinated? How many doses have you had? If not, why? If only one dose, why? |
| 1. From your point of view, how does your ability in finding online information shape your COVID-19 preventive behaviours? | - How online information may influence your COVID-19 preventive behaviours? - How about the influence of hoaxes on COVID-19 preventive behaviours? - What makes it difficult in applying COVID-19 preventive behaviours? - What makes it easy in applying COVID-19 preventive behaviours? |
